# A neural network approach to multi-biomarker panel discovery by high-throughput plasma proteomics profiling of breast cancer

**DOI:** 10.1186/1753-6561-7-S7-S10

**Published:** 2013-12-20

**Authors:** Fan Zhang, Jake Chen, Mu Wang, Renee Drabier

**Affiliations:** 1 Department of Academic and Institutional Resources and Technology, University of North Texas Health Science Center, Fort Worth, USA; 2 Department of Forensic and Investigative Genetics, University of North Texas Health Science Center, Fort Worth, USA; 3 School of Informatics, Indiana University, Indianapolis, IN 46202, USA; 4 Department of Computer and Information Science, School of Science, Purdue University, Indianapolis, IN 46202, USA; 5 Indiana Center for Systems Biology and Personalized Medicine, Indianapolis, IN 46202, USA; 6 Department of Biochemistry and Molecular Biology, Indiana University School of Medicine, USA; 7 Institute of Biopharmaceutical Informatics and Technology, Wenzhou Medical College, Wenzhou, Zhejiang, China

## Abstract

**Background:**

In the past several years, there has been increasing interest and enthusiasm in molecular biomarkers as tools for early detection of cancer. Liquid chromatography tandem mass spectrometry (LC/MS/MS) based plasma proteomics profiling technique is a promising technology platform to study candidate protein biomarkers for early detection of cancer. Factors such as inherent variability, protein detectability limitation, and peptide discovery biases among LC/MS/MS platforms have made the classification and prediction of proteomics profiles challenging. Developing proteomics data analysis methods to identify multi-protein biomarker panels for breast cancer diagnosis based on neural networks provides hope for improving both the sensitivity and the specificity of candidate cancer biomarkers for early detection.

**Results:**

In our previous method, we developed a Feed Forward Neural Network-based method to build the classifier for plasma samples of breast cancer and then applied the classifier to predict blind dataset of breast cancer. However, the optimal combination C* in our previous method was actually determined by applying the trained FFNN on the testing set with the combination. Therefore, in this paper, we applied a three way data split to the Feed Forward Neural Network for training, validation and testing based. We found that the prediction performance of the FFNN model based on the three way data split outperforms our previous method and the prediction performance is improved from (AUC = 0.8706, precision = 82.5%, accuracy = 82.5%, sensitivity = 82.5%, specificity = 82.5% for the testing set) to (AUC = 0.895, precision = 86.84%, accuracy = 85%, sensitivity = 82.5%, specificity = 87.5% for the testing set).

**Conclusions:**

Further pathway analysis showed that the top three five-marker panels are associated with complement and coagulation cascades, signaling, activation, and hemostasis, which are consistent with previous findings. We believe the new approach is a better solution for multi-biomarker panel discovery and it can be applied to other clinical proteomics.

## Introduction

Breast cancer is the most common cancer among American women, except for skin cancers. About 1 in 8 (12%) women in the US will develop invasive breast cancer during their lifetime. In 2012, an estimated 226, 870 new cases of invasive breast cancer were expected to be diagnosed in women in the U.S., along with 63,300 new cases of non-invasive (in situ) breast cancer [1].

In recent years, functional genomics studies using DNA Microarrays have been shown effective in differentiating between breast cancer tissues and normal tissues by measuring thousands of differentially expressed genes simultaneously [2-4]. However, early detection and treatment of breast cancer is still challenging. One reason is that obtaining tissue samples for microarray analysis can still be difficult. Another reason is that genes are not directly involved in any physical functions. On the contrary, the proteome are the real functional molecules and the keys to understanding the development of cancer. Moreover, the fact that breast cancer is a complex disease where disease genes exhibit an increased tendency for their protein products to interact with one another [5,6], makes the disease difficult to detect in early stages by single-marker approach. A chance of success with a multi-biomarker panel is higher than the simpler conventional single-marker approach [6].

Recent advances in clinical proteomics technology, particularly liquid chromatography coupled tandem mass spectrometry (LC-MS/MS) have enabled biomedical researchers to characterize thousands of proteins in parallel in biological samples. Using LC-MS/MS, it has become possible to detect complex mixtures of proteins, peptides, carbohydrates, DNA, drugs, and many other biologically relevant molecules unique to disease processes [7]. A modern mass spectrometry (MS) instrument consists of three essential modules: an ion source module that can transform molecules to be detected in a sample into ionized fragments, a mass analyzer module that can sort ions by their masses, charges, or shapes by applying electric and magnetic fields, and a detector module that can measure the intensity or abundance of each ion fragment separated earlier. Tandem mass spectrometry (MS/MS) has additional analytical modules for bombarding peptide ions into fragment peptide ions by pipelining two MS modules together, therefore providing peptide sequencing potentials for selected peptide ions in real time. LC-MS/MS proteomics has been used to identify candidate molecular biomarkers in a diverse range of samples, including cells, tissues, serum/plasma, and other types of body fluids. Due to the inherent high variability of both clinical samples and MS/MS instruments, it is still challenging to classify and predict proteomics profiles without an advanced computational method.

Developing a proteomics data analysis method to identify multi-protein biomarker panels for breast cancer diagnosis based on neural networks, therefore, provides hope for improving both the sensitivity and the specificity of candidate disease biomarkers. Neural Networks have several unique advantages and characteristics as research tools for cancer prediction problems [8-12]. A very important feature of these networks is their adaptive nature, where "learning by example" replaces conventional "programming by different cases" in solving problems [13].

The classification problem of breast cancer can be restricted to consideration of the two-class problem without loss of generality (breast cancer and normal). In the early case study [13], we developed a Feed Forward Neural Network-based method to build the classifier for plasma samples of breast cancer and then applied the classifier to predict blind dataset of breast cancer. However, the optimal combination C* was actually determined by applying the trained Feed Forward Neural Network (FFNN) on the testing set with the combination, which maximizes the AUC. Therefore, in this paper, we applied a three way data split to the FFNN for training, validation and testing based. Our results show the prediction performance of the FFNN model based on the three way data split outperforms our previous method in the earlier study [13].

We present the multi-marker panel development solution for early detection of breast cancer using the FFNN model based on three way data split, and show how to use it to model the classification and prediction problem of early detection of breast cancer in plasma proteomics.

## Materials and methods

### Human plasma samples

**Figure 1:**
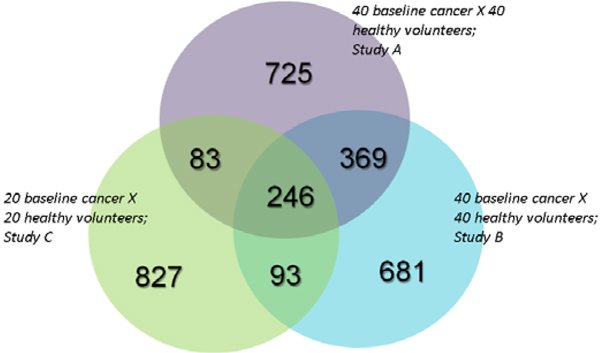
**Venn diagram of LC-MS/MS results**.

Three batches of plasma samples were collected by the Hoosier Oncology Group (HOG) (Indianapolis, IN, USA), which we call Study A, Study B, and Study C. Study A and Study B each contain 40 plasma samples from women with breast cancer and 40 plasma samples from healthy age-matched volunteer women as control. Study C collects 40 plasma samples with 20 samples from women diagnosed with breast cancer and 20 from healthy volunteer woman. All samples in the three studies were collected with the same standard operating procedure and stored in a central repository in Indianapolis, IN, USA. Study A, B and C were processed in the same laboratory but at different times. Each sample was analyzed in a single batch by mass spectrometry. The demography and clinical distribution of breast cancer stages for study A and B and C are comparable (Table 1), although the total sample number of Study C is somewhat smaller than Study A and B. For example, all patients involved in the three studies were diagnosed with a stage II or earlier breast cancer. Figure 1 indicates the degree of overlap among the proteins identified from three data sets: Study A, B and C.

**Table 1:** Comparison of clinical distribution for study A and B and C

	Study A	Study B	Study C
Cancer type	30 INV	23 INV	15 INV
	10 DCIS	8 DCIS	5 DCIS
		9 unknown	
ER+/PR+	29	23	12
ER- PR- HER2+	11	17	8
ER-/PR-/HER2-			

### LC/MS/MS plasma proteomics analysis

**Figure 2:**
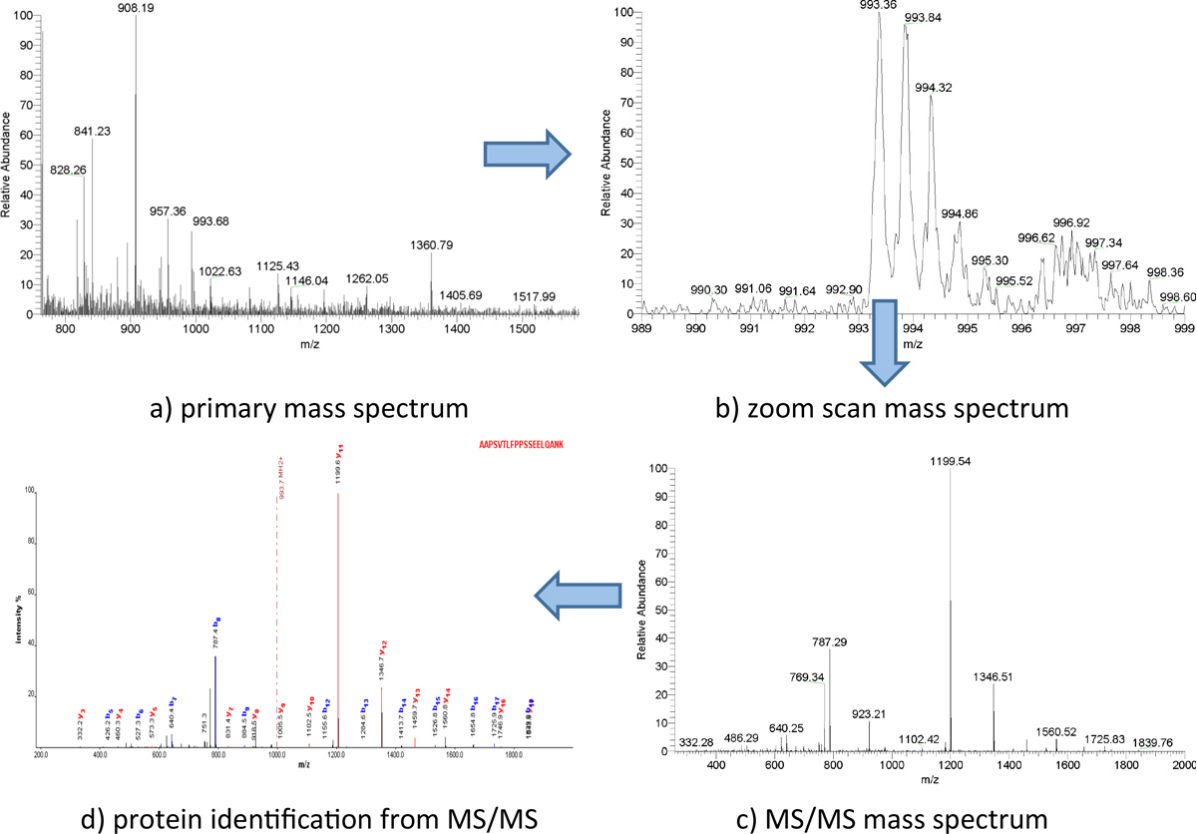
**Triple play mode (a) primary mass spectrum; b) zoom scan mass spectrum; c) MS/MS mass spectrum and d) protein identification from MS/MS)**.

Tryptic peptides were analyzed using Thermo-Fisher Scientific linear ion-trap mass spectrometer (LTQ) coupled with a Surveyor HPLC system (Thermo-Fisher Scientific) to identify proteins. Peptides were first eluted with a gradient from 5 to 45% Acetonitrile developed over 120 minutes at a flow rate of 50 μL/min. Data were collected in the *triple-play *mode (for example a) primary MS scan, b) zoom scan, and c) MS/MS scan in Figure 2) [14]. Lastly, the raw peak list data were generated by XCalibur (version 2.0) using default parameters and further analyzed by a label-free identification and quantitative algorithm using default parameters described by Higgs *et al *[15].

We performed the MS database search against the International Protein Index (IPI, version 3.6, Figure 2d) [16]. Protein quantification was carried out using the same algorithm mentioned before [15]. Briefly, first all extracted ion chromatograms (XIC) were aligned by retention time. Each aligned peak was matched by parent ion, charge state, daughter ions (MS/MS data) and retention time within a one-minute window. Then, the area-under-the-curve (AUC) for each individually aligned peak was measured, normalized, and compared for their relative abundance using methods described in [14,15].

### Linear mixed model

After transformation to a log_2 _scale and quantile normalization [17], the protein intensity is the final quantity that is fit by a separate analysis of variance (ANOVA) statistical model for each protein as *y_ijk _*using the following:

$$y_{ijk}=\mu +T_{j}+S_{k}+I_{i}+\varepsilon_{ijk},$$

where $I_{i}\sim N\left(0,\sigma_{1}^{2}\right),S_{k}\sim N\left(0,\sigma_{2}^{2}\right),\varepsilon_{jk}\sim N\left(0,\sigma^{2}\right)$ Here, *μ *is the mean intensity value, *T_j _*is the fixed group effect (caused by the experimental conditions or treatments being evaluated), *S_k _*is the random sample effect (random effects from either individual biological samples or sample preparations), *I_i _*is the random replicate effect (random effects from replicate injections of the same sample), and *ε_ijk _*is the within-groups errors. All of the injections were in random order and the instrument was operated by the same operator. All random effects are assumed independent of each other and independent of the within-group errors *ε_ijk_*.

### Feed forward neural network

**Figure 3:**
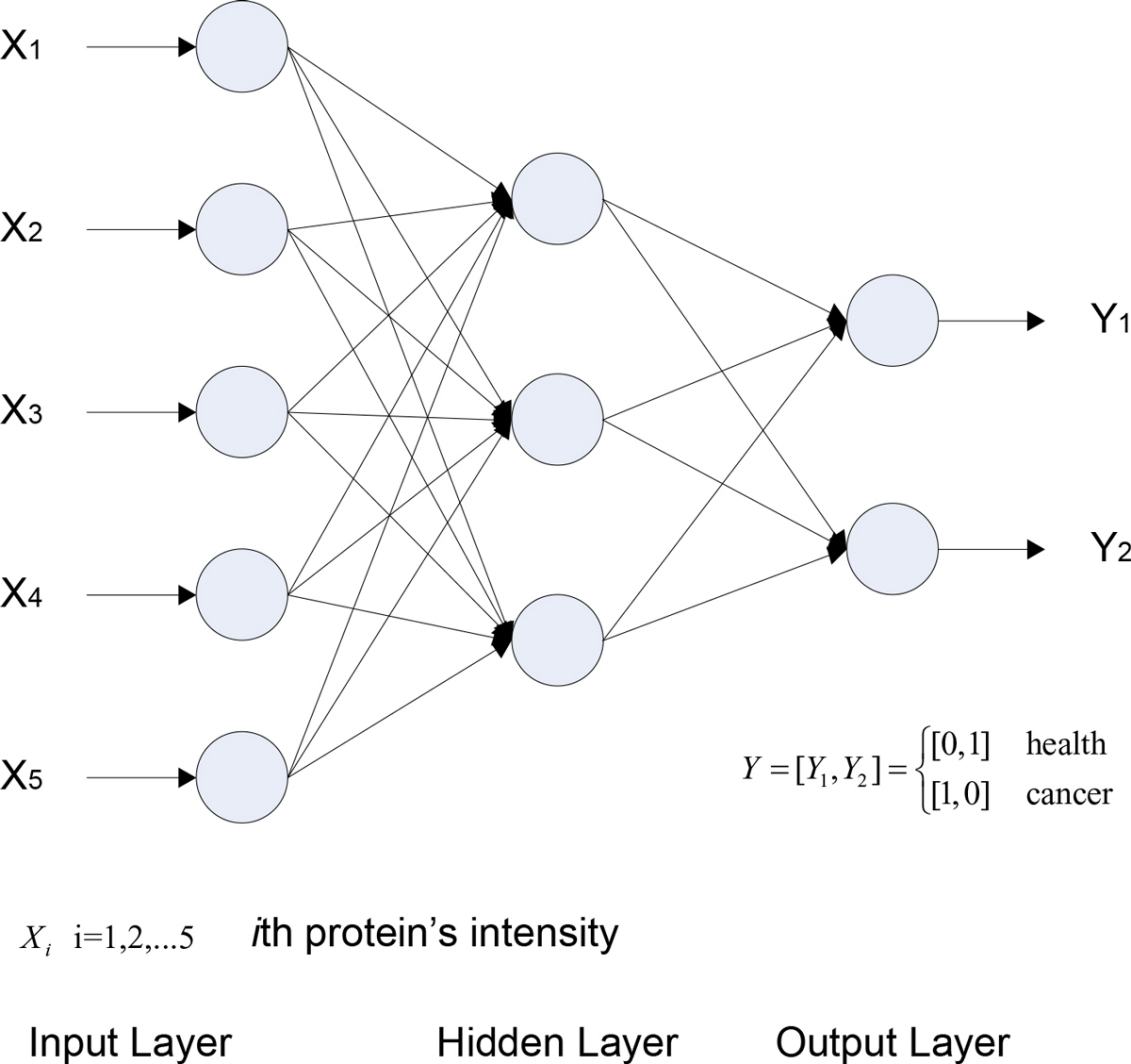
**Feed forward neural network for five-biomarker panel**.

A generalized Feed Forward Neural Network (FFNN) has three layers: input layer, hidden layer, and output layer and is trained using a back propagation supervised training algorithm. The input is used as activation for the input layer and is propagated to the output layer. The received output is then compared to the desired output and an error value is calculated for each node in the output layer. The weights on edges going into the output layer are adjusted by a small amount relative to the error value. This error is propagated backwards through the network to correct edge weights at all levels. For example, Figure 3 described a feed forward neural network with an input layer of 5 nodes (corresponding to a five-marker panel), a hidden layer of 3 nodes, and an output layer of two-variable encoding scheme [healthy = (0,1), cancer = (1,0)]. We used empirically-derived rules-of-thumb, the most commonly relied on, which is 'the optimal size of the hidden layer is usually between the size of the input and size of the output layers' [18] to determinate the size of hidden layer.

The classification problem of breast cancer can be restricted to consideration of the two-class problem without loss of generality (breast cancer and normal). An FFNN-based method [13] was used to develop the classifier for plasma samples of breast cancer and then applied the classifier to predict blind dataset of breast cancer. Based on the FFNN-based method, however, we apply in the paper a three way data split for training, validation and testing, instead of directly using Study A as a training set and Study B as a testing set in the [13]. Briefly, Study A is used as the training set for learning to fit the parameters of the classifier, Study C as the validation set to tune the parameters of the classifier, and Study B as the testing set only to assess the performance of the fully-trained classifier.

The enumeration method based on FFNN was built to identify optimal biomarkers panel by us [13]. Similarly, we designed an enumeration method based on the three way data split and the FFNN to find the optimal classifier, which measures the area under the curve (AUC) for Receiver Operating Characteristics (ROC).

Each combination of N (N = 5 for five-marker panel) out of all the 32 genes differentially expressed in the training set is chosen as inputs to the FFNN. We select N = 5 because 1) Li estimated that five or six genes rather than 37 or 738 would be sufficient for the early detection of breast cancer, based on colon cancer, leukemia, and breast cancer [19], 2) we expect to achieve high prediction accuracy for breast cancer with as few genes as possible, and 3) we applied N = 5 to the prediction of breast cancer in our previous study and achieved satisfied prediction performance [13].

In this scheme, we first train the FFNN for each combination in the training set with 5-fold cross-validation. Then, we measure the AUC for each combination in the validation set. Lastly, the optimal combination C* was determined by

$$C^{*}={\text{argmax}}_{C}AUC\left(NET_{C},V\right),$$

where AUC is the area under the ROC curve of the FFNN, C is combination of picking five out of the 32 genes, and V is the validation set.

### Pathway analysis

Pathway analysis is performed using the following databases: Integrated Pathway Analysis Database (IPAD) http://bioinfo.hsc.unt.edu/ipad/[20].

## Results and discussions

The plasma proteome sets from Study A, B, and C contains 1423, 1389, and 1249 proteins, respectively. 246 proteins are in common between the three datasets. After ANOVA analysis of the 246 proteins in the Study A, we obtained 32 candidate markers in the training set with pvalue < 0.01. No data from the testing set were utilized in 1) identification of breast cancer markers or 2) development of the FFNN model. The validation set was used to tune the parameters of the FFNN model.

Based on an FFNN model that was built on all 60 markers differentially expressed in Study A and Study B, a high performance (AUC = 0.8713, precision = 86.8%, accuracy = 85%, sensitivity = 82.5%, specificity = 87.5% for the training set; AUC = 0.8706, precision = 82.5%, accuracy = 82.5%, sensitivity = 82.5%, specificity = 82.5% for the testing set) was obtained [13]. However, the optimal combination C* was actually determined by applying the trained FFNN on the testing set with the combination, which maximizes the AUC. This step obtained an objective optimization with training set and testing set. Therefore, in this paper, we applied the three way data split for training, validation and testing and constructed an FFNN for each combination of five out of the 32 markers and trained with plasma samples derived from 40 women diagnosed with breast cancer and 40 control women in the training set. The optimal combinations were obtained by our optimization model based on the training set and validation set instead of the training set and testing set we used in [13].

Training of the FFNN was performed using back propagation algorithm for two-variable encoding scheme, because we had verified that the two-variable encoding scheme performed better than single-variable encoding scheme [13]. Five performance measurements: (1) Sensitivity; (2) Specificity; (3) Precision; (4) Accuracy; and (5) Area Under the Curve were computed in order to evaluate the prediction performance of the FFNN.

**Figure 4:**
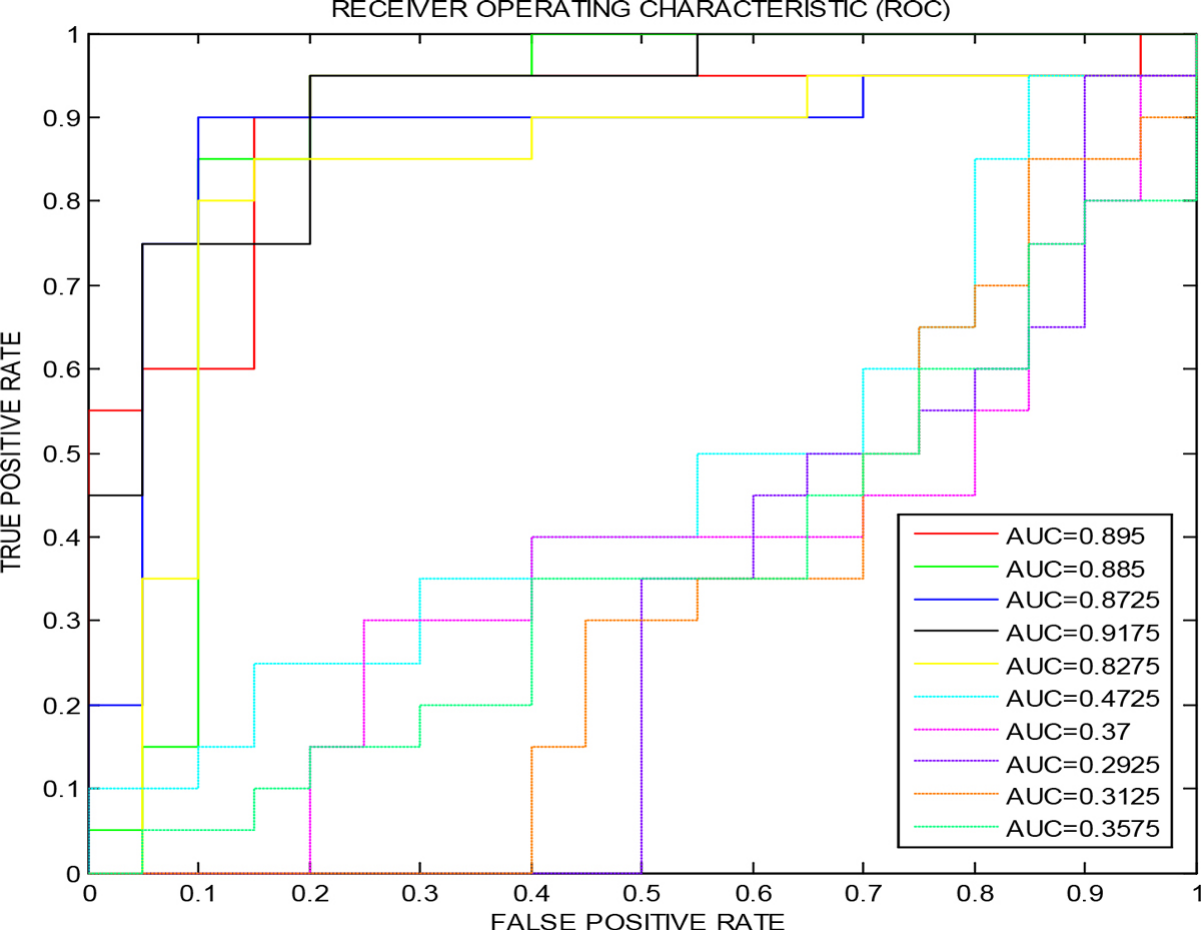
**A comparison of best five 5-marker panel ROCs (solid lines) and randomly chosen five (out of 32 candidates) 5-marker ROCs (dotted lines)**.

In order to validate our prediction method, we compared the ROCs for the best five 5-marker panel predictions determined by our method with the ROCs for five randomly chosen 5-marker panels from 32 candidate biomarkers (Figure 4). As shown in the Figure 4, the top five best predictions determined by our method (solid lines) has better sensitivity-specificity-tradeoff performance than those chosen randomly from 32 candidate biomarkers.

Table 2 shows the best three five-marker panels identified using the FFNN and the three way data split. Gene C4BPA is in common between the best three five-marker panels and gene SRCRB4D shows up twice.

**Table 2:** Best three five-marker panels identified

Panel	SSE1	Accuracy		
		
		Training Set	Testing Set	Validation Set
C4BPA; HP; ORM1; SAMD9; SRCRB4D	3.3E-2	0.875	0.85	0.85

C4BPA; STBD1; DDX24; GRASP; CFI	5.6E-2	0.875	0.8375	0.85

C4BPA; CNO; FGG; SERPING1; SRCRB4D	1.9E-2	0.8625	0.85	0.85

Pathway analysis shows the pathways linked with the best three five-marker panels are complement and coagulation cascades, signaling, activation, and hemostasis (Table 3), which are consistent with previous results we found [6]. The confusion matrix and common performance metrics for the training set, testing set, and validation set for the best five-marker panel is shown in the Table 4. Although the final sensitivity is 82.5%, the same as previous result in [13], the other performance measures are improved. For example, the final accuracy increases from 82.5% to 85% and the final specificity from 82.5% to 87.5% (Table 4). In addition, the AUC, a comprehensive measurement of sensitivity and specificity, is improved from 0.8706 to 0.895 (Figure 4). The prediction performance of the FFNN model based on three way data split (Study A as training set, Study B as testing set, and Study C as validation set ) actually outperforms our previous method in [13]. For example, the specificities are both 87.5% for the training sets. But after using the validation set to tune the parameter of the model, the final specificity for the testing set is improved from 82.5% to 87.5%.

**Table 3:** Pathway analysis for the best three five-marker panels.

PathwayID	PathwayName	Molecule
hsa04610	Complement and coagulation cascades	FGG;SERPING1;C4BPA;CFI
h_intrinsicPathway	Intrinsic Prothrombin Activation Pathway	FGG;SERPING1
140877	Formation of Fibrin Clot (Clotting Cascade)	FGG;SERPING1
114608	Platelet degranulation	SERPING1;FGG
76005	Response to elevated platelet cytosolic Ca2+	SERPING1;FGG
hsa05133	Pertussis	SERPING1;C4BPA
hsa05150	Staphylococcus aureus infection	FGG;CFI
354194	GRB2:SOS provides linkage to MAPK signaling for Intergrins	FGG
140875	Common Pathway	FGG
76002	Platelet activation, signaling and aggregation	FGG;SERPING1
372708	p130Cas linkage to MAPK signaling for integrins	FGG
h_extrinsicPathway	Extrinsic Prothrombin Activation Pathway	FGG
h_fibrinolysisPathway	Fibrinolysis Pathway	FGG
140837	Intrinsic Pathway	SERPING1
h_amiPathway	Acute Myocardial Infarction	FGG
200138	Beta2 integrin cell surface interactions	FGG
200204	Ephrin B reverse signalling	FGG
200037	CD40/CD40L signalling	C4BPA
354192	Integrin alphaIIb beta3 signaling	FGG
76009	Platelet Aggregation (Plug Formation)	FGG
200043	Beta3 integrin cell surface interactions	FGG
200061	Regulation of RhoA activity	HP
200139	Urokinase-type plasminogen activator (uPA) and uPAR-mediated signalling	FGG
200146	IL6-mediated signaling events	FGG
hsa05143	African trypanosomiasis	HP
432722	Golgi Associated Vesicle Biogenesis	CNO
109582	Hemostasis	FGG;SERPING1
199992	trans-Golgi Network Vesicle Budding	CNO
421837	Clathrin derived vesicle budding	CNO
200016	Beta1 integrin cell surface interactions	FGG

**Table 4:** Prediction result for the best 5-marker panel

Predicted	Training Set			Testing Set			Validation Set		
	Cancer	Normal		Cancer	Normal		Cancer	Normal	
Cancer	35		5	33		5	17		3
Normal	5		35	7		35	3		17
Precision		87.5%			86.84%			85%	
Accuracy		87.5%			85%			85%	
Sensitivity		87.5%			82.5%			85%	
Specificity		87.5%			87.5%			85%	

We also compared the three way data split with other combination such as mixing three datasets. When mixing three datasets together, the best marker panel has the same performance in the training mode (precision = 87.5%, accuracy = 87.5%, sensitivity = 87.5%, specificity = 87.5%), and a little higher performance in the testing mode (Study B as testing set, precision = 87.18%, accuracy = 86.25%, sensitivity = 85%, specificity = 87.5%). The reason why the performance is higher in the testing mode is because the mixture of three datasets already contains the testing set and the testing set is not independent of the training set. The three way data split method is more close to real applications where testing data are blind or unknown. The prediction performance of the testing set in a three-way data split can better reflect the outcome in a real application than other combination such as the mixture of three datasets. We believe the new approach is a better solution for multi-biomarker panel discovery and it can be applied to other clinical proteomics.

## Conclusions

We developed a Feed Forward Neural Network approach that addressed a challenging multi-panel biomarker development problem in the early detection of breast cancer. The approach that we used combined the three way data split with an optimization model of FFNN. We found that the prediction performance of the FFNN model combined with the three way data split outperforms our previous method. Further pathway analysis showed that the top three five-marker panels are associated with complement and coagulation cascades, signaling, activation, and hemostasis, which are consistent with previous findings. We believe the new method is a better solution for multi-biomarker panel discovery and can provide general guidance for future molecular medicine multi-marker panel discovery applications in other diseases. In the future, we will follow up with biological experiments to validate these biomarkers with our collaborators.

## Competing interests

The authors declare that they have no competing interests.

## Authors' contributions

RD and FZ conceived the initial work and designed the method. FZ and RD developed the prediction method, and performed the computational analyses. MW and JC provided experimental data. All authors are involved in the drafting and revisions of the manuscript.

## Declarations

Publication of this work was supported by the Bioinformatics Program at University of North Texas Health Science Center.

This article has been published as part of *BMC Proceedings *Volume 7 Supplement 7, 2013: Proceedings of the Great Lakes Bioinformatics Conference 2013. The full contents of the supplement are available online at http://www.biomedcentral.com/bmcproc/supplements/7/S7.
